# Integrated Phytochemical Profiling and Multifunctional Bioactivities of *Bellis annua* Extracts Obtained by Distinct Extraction Techniques

**DOI:** 10.1002/open.202500603

**Published:** 2026-04-21

**Authors:** Fatma Ozlem Kargin Solmaz, Cengiz Sarikurkcu, Bektaş Tepe

**Affiliations:** ^1^ Faculty of Pharmacy Afyonkarahisar Health Sciences University Afyonkarahisar Türkiye; ^2^ Faculty of Science Department of Molecular Biology and Genetics Kilis 7 Aralik University Kilis Türkiye

**Keywords:** antioxidant activity, *Bellis annua*, enzyme inhibition, extraction techniques, phytochemical composition

## Abstract

This study presents an integrated chemical and biological assessment of *Bellis annua* methanolic extracts obtained through maceration (MAC), Soxhlet extraction (SOE), and ultrasound‐assisted extraction (UAE). LC–electrospray ionization‐mass spectrometry (ESI–MS)/MS profiling enabled the quantification of major phenolic compounds, including chlorogenic acid, hyperoside, hesperidin, and several hydroxycinnamic acids, revealing extraction‐dependent variations in phenolic distribution. While total phenolic levels were comparable across methods, UAE yielded the highest flavonoid content and a broader enrichment of glycosylated flavonoids and hydroxycinnamates. Antioxidant capacity was evaluated using 2,2‐diphenyl‐1‐picrylhydrazyl (DPPH) and 2,2′‐azino‐bis(3‐ethylbenzothiazoline‐6‐sulfonic acid) (ABTS) radical‐scavenging assays, ferric reducing antioxidant power, cupric reducing antioxidant capacity, the phosphomolybdenum method, and a metal‐chelation assay. All assays indicated notable activity of the extracts. UAE exhibited the strongest overall antioxidant performance as indicated by the Relative Antioxidant Capacity Index. Enzyme inhibition assays revealed method‐specific differences: MAC showed the most pronounced acetylcholinesterase and *α*‐amylase inhibition, whereas SOE demonstrated superior tyrosinase inhibition. Correlation analyses further indicated that flavonoid‐rich extracts were strongly associated with radical‐scavenging and metal‐chelating activities, while specific phenolic acids contributed differentially to enzyme inhibition patterns. These findings highlight the influence of extraction technique on the chemical composition and multifunctional bioactivity of *B. annua*, supporting its potential as a promising natural source of phenolic‐driven antioxidant and enzyme‐inhibitory agents with relevance to pharmaceutical and nutraceutical applications.

## Graphical abstract

The present study evaluates the effect of maceration, Soxhlet, and ultrasound‐assisted extraction on the phytochemical composition and biological activities of Bellis annua. LC–ESI–MS/MS analysis revealed chlorogenic acid as the dominant compound, while UAE enhanced flavonoid content and overall antioxidant capacity. Extraction‐dependent variations were also observed in enzyme inhibition profiles.

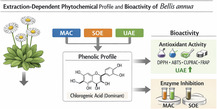

## 1 Introduction

Phenolic compounds derived from plants play important roles in maintaining oxidative balance and regulating enzymatic processes in biological systems due to their redox properties, metal‐chelating capacity, and ability to scavenge reactive oxygen species [1]. Therefore, studies aimed at discovering biologically effective molecules from natural sources are increasingly being conducted. The antioxidant capacity of plant extracts should be assessed using multiple methods reflecting different mechanisms of action, as no single analysis can reveal all the redox behavior of complex mixtures. One method used for this purpose, CUPRAC (cupric reducing antioxidant capacity), stands out for its ability to measure reducing power at pH levels close to physiological conditions. The CUPRAC method is frequently applied in conjunction with 2,2‐diphenyl‐1‐picrylhydrazyl (DPPH) and 2,2′‐azino‐bis(3‐ethylbenzothiazoline‐6‐sulfonic acid) (ABTS) radical‐scavenging assays, ferric reducing antioxidant power (FRAP), the phosphomolybdenum assay, and ferrous ion chelation tests, providing a holistic view of the antioxidant potential of plant extracts [2, 3]. Among antioxidant assays, the oxygen radical absorbance capacity method is also widely used, particularly for evaluating hydrogen atom transfer–based radical scavenging activity in complex biological matrices [4].

In addition to their antioxidant properties, phenolic compounds are also well known to exhibit inhibitory effects against a range of biologically relevant enzymes, thereby contributing to their therapeutic potential [5, 6]. For example, inhibition of acetylcholinesterase (AChE) is one of the accepted strategies in the symptomatic treatment of Alzheimer's disease and provides temporary cognitive improvement by increasing synaptic acetylcholine levels [6]. Similarly, inhibition of *α*‐amylase helps control postprandial hyperglycemia by slowing the rate of carbohydrate digestion, an effect that has long been evaluated in the treatment of type 2 diabetes [7]. In the cosmetics and food industries, inhibition of tyrosinase is important for reducing skin hyperpigmentation and preventing undesirable browning in fruit and vegetable products [8]. Therefore, when both the chemical profiles and biological activities of plant extracts are examined together, a deeper understanding of their functional potential can be obtained.

*Bellis annua* *L.* (Asteraceae) is an annual herbaceous species whose native range extends from the Canary Islands through the Mediterranean region to Iran. Within the genus *Bellis*, considerable phytochemical and ethnomedicinal research has focused on *B. perennis* [9, 10], whereas comparable information for *B. annua* remains scarce. Compared to other members of the genus *Bellis*, this species has been poorly investigated from a phytochemical perspective. Only limited phytochemical information is available, mainly concerning the identification of triterpenoid saponins, while data on its volatile composition have been lacking. Recently, the chemical composition of the essential oils obtained from different aerial parts of *B. annua* collected in Sicily was reported for the first time, revealing a profile dominated by monoterpene hydrocarbons, particularly *β‐myrcene* in the flowers and hexadecanal in the leaves and stems. These findings highlight the limited current knowledge on *B. annua* *a*nd support further investigations into its chemical composition and biological potential [11]*.*

The extraction approach plays a decisive role in the component diversity and biological activity of an extract. While conventional methods such as maceration (MAC) and Soxhlet extraction (SOE) maintain their importance in terms of reliability, they have drawbacks such as long processing times and high solvent consumption [12]. In contrast, ultrasound‐assisted extraction (UAE) accelerates mass transfer by increasing solvent penetration through acoustic cavitation, often resulting in improved extraction efficiency and better preservation of heat‐sensitive phenolic compounds [13, 14]. This method is particularly advantageous for the preservation of heat‐sensitive phenolics. Numerous studies in the literature emphasize that UAE is a more effective and environmentally friendly alternative to traditional methods; however, this efficiency may vary depending on factors such as solvent type, solid/liquid ratio, ultrasound power, and matrix properties [13, 15, 16, 17]. To enable a reliable comparison among extraction techniques, the solvent variable was intentionally kept constant, and methanol was selected as a model solvent due to its well‐documented efficiency in extracting a broad range of phenolic compounds.

Methanol is one of the most commonly used solvents in the extraction of phenolic compounds from plant materials due to its high polarity and efficiency in solubilizing a wide range of antioxidant constituents. Several studies have demonstrated that methanolic extracts yield higher total phenolic content and stronger antioxidant activity compared to less polar solvents, justifying its frequent selection in phytochemical and antioxidant investigations [18, 19]. In the context of the present study, the use of methanol was therefore considered appropriate to ensure efficient recovery of phenolic constituents from *B. annua* and to allow a reliable comparison of extraction techniques.

For these reasons, (i) comparison of different extraction techniques, (ii) determination of total phenolic and flavonoid content, (iii) identification of selected phenolic compounds by LC‐electrospray ionization‐mass spectrometry (ESI‐MS)/MS, and (iv) conducting multiple antioxidant and enzyme inhibition tests allow for a holistic elucidation of the relationships between the chemical structure and biological activity of *B. annua*. In the present study, optimization does not refer to the adjustment of extraction parameters or solvent systems but rather to a comparative evaluation of different extraction techniques performed under fixed solvent conditions in order to assess their influence on phenolic composition and biological activity. LC–ESI–MS/MS is a well‐established and widely applied analytical technique for the sensitive and selective targeted quantification of phenolic compounds in complex plant matrices [20]. Furthermore, calculation of the RACI (relative antioxidant capacity index) value to eliminate scale differences in the results obtained from different antioxidant tests allows for a more statistically significant interpretation of the data [21].

In this study, methanolic extracts obtained from the aerial parts of *B. annua* by MAC, UAE, and Soxhlet (SOE) methods were evaluated. Total phenolic and flavonoid contents of the obtained extracts and their LC‐ESI‐MS/MS profiles were correlated with antioxidant activities determined by phosphomolybdenum, ferrous ion chelation, DPPH, ABTS, CUPRAC, and FRAP tests. Furthermore, inhibitory activities against AChE, *α‐*amylase, and tyrosinase were investigated; the results were supported by RACI and correlation analyses. Thus, the versatile biological potential of *B. annua*, originating from its phenolic composition, was elucidated, and it was demonstrated that this species can be considered among the natural sources of antioxidants and enzyme inhibitors.

## 2 Material and Methods

### 2.1 Plant Material

The aerial parts of *B. annua* were collected on 3 April 2023 from Salkım Village, Kavaklıdere, Muğla, Türkiye (800 m a.s.l., 37°30′63″ N, 28°17′26″ E). The species was taxonomically authenticated by Dr. Olcay CEYLAN (Department of Biology, Muğla Sıtkı Koçman University, Muğla, Türkiye), and a voucher specimen was deposited in the university herbarium under the number O.1251. After collection, the aerial parts were cleaned and dried under shade at ambient temperature with adequate air circulation until a constant weight was achieved. The dried material was then ground into a fine powder and stored in airtight containers protected from light and moisture until extraction.

### 2.2 Preparation of Methanol Extracts

Three different extraction methods were employed to obtain methanolic extracts of the plant material following the procedure of Zengin et al. [22]. For MAC, 5 g of air‐dried powdered sample was soaked in 100 mL of methanol and agitated for 24 h at room temperature. UAE was conducted for 1 h in a sonication bath using the same solvent and a sample‐to‐solvent ratio of 1:20. SOE was carried out with methanol for 6 h using a conventional Soxhlet apparatus. All obtained extracts were filtered and concentrated to dryness under reduced pressure at 40°C, then stored at 4°C in the dark until further analyses. After solvent evaporation, the dried extracts were redissolved in methanol to prepare stock solutions. Appropriate working dilutions were then prepared according to each assay requirement. For spectrophotometric analyses, including the Folin–Ciocalteu method, the methanolic stock solutions were suitably diluted to avoid solvent‐related interference or precipitation.

### 2.3 Determination of the Phenolic Composition

Total phenolic and flavonoid contents of the extracts were quantified spectrophotometrically as described by Zengin et al. [23]. Qualitative and quantitative profiling of selected phytochemicals was carried out using a previously validated LC–ESI–MS/MS method [20], with the main analytical parameters summarized in Tables S1 and S2 of the Supporting Information. The analysis was conducted using a targeted multiple reaction monitoring approach based exclusively on authentic phenolic standards. Consequently, secondary metabolites outside the predefined *m*/*z* transitions of the selected phenolic compounds were not evaluated within the scope of this study. Quantification was achieved using external calibration curves constructed with the corresponding standards. An internal standard was not employed, as the method had been previously validated for targeted phenolic analysis under stable instrumental conditions, and quantitative reliability was ensured through calibration linearity and replicate injections.

### 2.4 Biological Activity Assays

Antioxidant activities of the extracts were evaluated using a series of complementary assays, including phosphomolybdenum, ferrous ion‐chelating, DPPH, ABTS, CUPRAC, and FRAP methods [1, 23, 24, 25, 26]. Enzyme inhibition studies targeting acetylcholinesterase, *α*‐amylase, and tyrosinase were carried out according to the procedure of Ozer et al. [27]. Further experimental details are provided in the supporting information

### 2.5 Statistical Analysis

The RACI was calculated following the approach of Sun and Tanumihardjo [21]. All statistical evaluations and correlation analyses were performed as described in the supporting information.

## 3 Results and Discussion

### 3.1 Chemical Compositions of the Extracts

**FIGURE 1:**
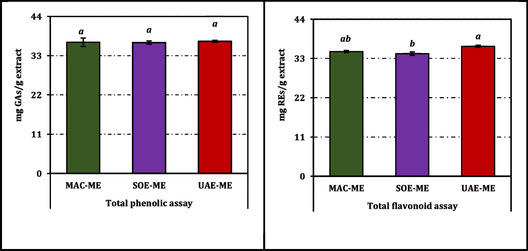
Total phenolic and flavonoid contents of the methanol extracts from *B. annua*. GAEs and REs: Gallic acid and rutin equivalents, respectively. Data are presented as mean ± standard deviation (SD) of three independent experiments (*n* = 3). Statistical differences were evaluated using one‐way ANOVA followed by Tukey's honestly significant difference (HSD) post hoc test at *p *< 0.05.

Total phenolics in the methanol extracts of *B. annua* were narrowly distributed across techniques, with MAC‐ME, SOE‐ME, and UAE‐ME yielding 36.76, 36.66, and 37.03 mg GAEs/g extract, respectively; no pairwise differences were detected (Figure 1). By contrast, total flavonoids showed a modest but significant technique effect: UAE‐ME (36.41 mg REs/g) > MAC‐ME (34.93 mg REs/g) ≥ SOE‐ME (34.35 mg REs/g), as indicated by distinct superscripts (Figure 1).

**FIGURE 2:**
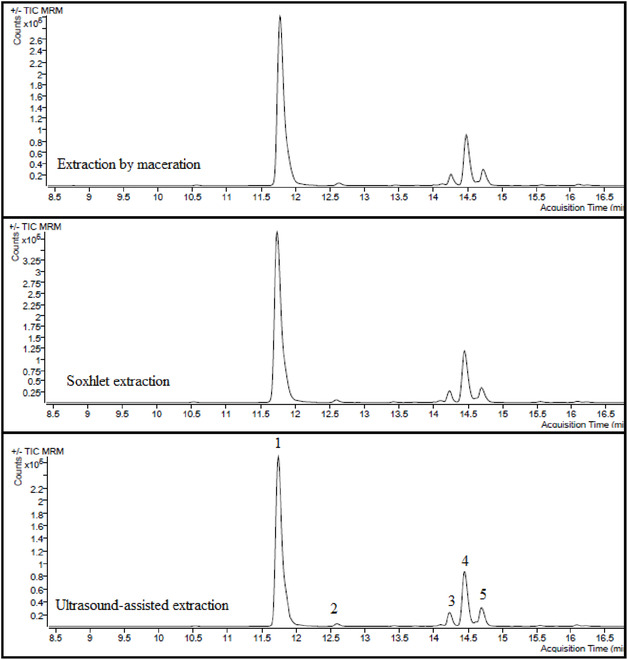
LC–ESI–MS/MS chromatograms of the methanol extracts from *B. annua* using different extraction techniques. 1: Chlorogenic acid, 2: Caffeic acid, 3: Luteolin 7‐glucoside, 4: Hesperidin and Hyperoside, and 5: Apigenin 7‐glucoside.

LC–ESI–MS/MS profiling confirmed closely overlapping qualitative fingerprints among the three extracts (Figure 2). Twenty‐four targeted phenolics were monitored, of which several were abundant while others were trace or absent (Table 1).

**TABLE 1:** Concentration (µg/g extract) of selected phenolic compounds in the methanol extracts from *B. annua*.

Compounds	MAC‐ME	SOE‐ME	UAE‐ME
Chlorogenic acid	15,877 ± 29^*a*^	14,200 ± 129^*b*^	13,986 ± 165^*b*^
Hyperoside	3649 ± 32^*a*^	3319 ± 54^*b*^	3594 ± 72^*a*^
Hesperidin	1456 ± 8^*a*^	1184 ± 33^*b*^	1503 ± 41^*a*^
Apigenin 7‐glucoside	712 ± 2^*b*^	584 ± 2^*c*^	744 ± 3^*a*^
Caffeic acid	175 ± 1^*a*^	154 ± 1^*b*^	173 ± 3^*a*^
Luteolin 7‐glucoside	156 ± 1^*b*^	137 ± 1^*c*^	183 ± 2^*a*^
Protocatechuic acid	124 ± 1^*a*^	116 ± 1^*b*^	120 ± 3^*ab*^
Kaempferol	120 ± 2^*b*^	86.7 ± 0.4^*c*^	137 ± 2^*a*^
Verbascoside	67.9 ± 1.1^*b*^	72.2 ± 0.6^*b*^	87.0 ± 2.2^*a*^
4‐Hydroxybenzoic acid	51.4 ± 0.3^*b*^	51.8 ± 0.6^*b*^	69.7 ± 0.6^*a*^
Apigenin	36.4 ± 0.2^*b*^	32.5 ± 0.3^*c*^	42.8 ± 1.0^*a*^
Quercetin	32.5 ± 1.7^*b*^	40.8 ± 1.4^*ab*^	43.2 ± 3.2^*a*^
Gallic acid	29.3 ± 0.1^*a*^	21.7 ± 0.1^*b*^	17.8 ± 0.5^*c*^
*p*‐Coumaric acid	17.4 ± 0.1^*b*^	19.6 ± 0.6^*a*^	20.9 ± 0.2^*a*^
Rosmarinic acid	16.6 ± 0.7^*b*^	16.2 ± 0.3^*b*^	20.0 ± 0.1^*a*^
Ferulic acid	16.3 ± 0.2^*b*^	15.6 ± 0.1^*b*^	19.6 ± 0.3^*a*^
Luteolin	14.5 ± 0.1^*b*^	13.4 ± 0.2^*b*^	19.5 ± 0.5^*a*^
2,5‐Dihydroxybenzoic acid	12.5 ± 0.2^*a*^	9.92 ± 0.12^*c*^	11.8 ± 0.1^*b*^
Syringic acid	9.65 ± 0.12^*a*^	8.48 ± 0.45^*a*^	8.22 ± 0.47^*a*^
Vanillin	4.61 ± 0.26^*c*^	8.52 ± 0.48^*a*^	6.73 ± 0.41^*b*^
Sinapic acid	2.45 ± 0.03^*b*^	2.21 ± 0.08^*c*^	2.96 ± 0.04^*a*^
(−)‐Epicatechin	0.56 ± 0.01^*b*^	0.51 ± 0.03^*b*^	0.93 ± 0.01^*a*^
Pinoresinol	nd	nd	nd
Pyrocatechol	nd	nd	nd
3,4‐Dihydroxyphenylacetic acid	nd	nd	nd
(+)‐Catechin	nd	nd	nd
3‐Hydroxybenzoic acid	nd	nd	nd
Taxifolin	nd	nd	nd
2‐Hydroxycinnamic acid	nd	nd	nd
Eriodictyol	nd	nd	nd

Data are presented as mean ± standard deviation (SD) of three independent experiments (*n* = 3). Statistical differences were evaluated using one‐way ANOVA followed by Tukey's HSD post hoc test at *p *< 0.05. nd: Not detected.

Identical superscript letters within the same column indicate that the corresponding values are not significantly different at the *p *< 0.05 level.

Quantitatively, chlorogenic acid dominated the profiles (≈14.000–15.900 µg/g extract), peaking in MAC‐ME (15.877 µg/g) and decreasing in SOE‐ME (14.200 µg/g) and UAE‐ME (13.986 µg/g). Among flavonol/flavone derivatives, hyperoside (3.319–3.649 µg/g) and hesperidin (1.184–1.503 µg/g) were consistently high; both were significantly enriched in MAC‐ME and/or UAE‐ME relative to SOE‐ME (Table 1). UAE‐ME generally favored higher levels of several glycosides and aglycones, including apigenin 7‐glucoside (744 µg/g), luteolin 7‐glucoside (183 µg/g), kaempferol (137 µg/g), apigenin (42.8 µg/g), and quercetin (43.2 µg/g), while SOE‐ME showed the lowest values for many of these markers. Notable exceptions included gallic acid, which was maximal in MAC‐ME (29.3 µg/g) and declined across SOE‐ME (21.7 µg/g) to UAE‐ME (17.8 µg/g), and vanillin, which was highest in SOE‐ME (8.52 µg/g). For hydroxycinnamates, UAE‐ME returned the greatest concentrations of *p*‐coumaric (20.9 µg/g), ferulic (19.6 µg/g), rosmarinic (20.0 µg/g), and sinapic acids (2.96 µg/g), whereas MAC‐ME led in caffeic acid (175 µg/g) and 2,5‐dihydroxybenzoic acid (12.5 µg/g). Syringic acid did not differ statistically among techniques, while protocatechuic acid was slightly higher in MAC‐ME than SOE‐ME, with UAE‐ME intermediate. (−)‐Epicatechin was detectable in all extracts and maximized in UAE‐ME (0.93 µg/g). Following analysis, it was determined that several analytes were not detected in any of the samples (Table 1). These analytes include pinoresinol, pyrocatechol, 3,4‐dihydroxyphenylacetic acid, (+)‐catechin, 3‐hydroxybenzoic acid, 2‐hydroxycinnamic acid, taxifolin, and eriodictyol. It should be noted that the applied LC–ESI–MS/MS method was designed as a targeted analysis of selected phenolic compounds, and other classes of secondary metabolites were not evaluated within this analytical framework.

Taken together, while the global phenolic load was comparable across techniques, UAE‐ME produced the highest total flavonoids and tended to enrich multiple flavonoid glycosides and aglycones, whereas MAC‐ME preferentially concentrated chlorogenic and gallic acids. All inferences are supported by Tukey's HSD at *α *= 0.05 (Table 1 and Figures 1 and 2).

The present investigation provides a targeted phenolic characterization of B. annua methanolic extracts based on the quantitative determination of selected compounds using LC–ESI–MS/MS. It should be noted that the analytical scope of the present study was limited to targeted compounds for which authentic standards were available, and no untargeted metabolomic screening was performed. LC–ESI–MS/MS analysis revealed a diverse phenolic profile dominated by chlorogenic acid and its derivatives, along with notable quantities of flavonoid glycosides such as hyperoside, hesperidin, and apigenin 7‐glucoside. This composition aligns partially with reports from other *Bellis* species, although certain compound distributions appear to be species‐specific or extraction‐dependent.

The predominance of chlorogenic acid in *B. annua* is consistent with findings in *B. sylvestris*, in which chlorogenic and neochlorogenic acids, together with other caffeoylquinic acid derivatives, were reported as major phenolics [28]. Similarly, *B. perennis* tissues were shown to accumulate chlorogenic and caffeic acids under specific culture conditions [29], supporting the notion that hydroxycinnamic acids represent a chemotaxonomic hallmark across the *Bellis* genus. The detection of rosmarinic acid in *B. annua* further reinforces this pattern, given their recurrence in the leaves of *B. sylvestris* [28] and the proposed allelopathic or defensive functions of caffeic acid derivatives within *Bellis* species [30]. Such metabolites are well known to participate in oxidative stress regulation, antimicrobial defense, and ecological interactions, indicating a conserved biochemical strategy among *Bellis* taxa.

The flavonoid composition of *B. annua*, particularly the enrichment of glycosylated flavonols (e.g., hyperoside, hesperidin, luteolin 7‐glucoside, and quercetin derivatives), mirrors that of *B. perennis*, where apigenin‐7‐*O*‐glucopyranoside and a range of quercetin and kaempferol glycosides were identified [9, 28]. These flavonoids are commonly implicated in photoprotection and ROS scavenging, which may explain their consistent accumulation across *Bellis* species exposed to different environmental pressures. The relatively higher levels of aglycones such as apigenin and kaempferol observed in the UAE‐ME extract suggest that ultrasonic energy may promote the cleavage of glycosidic bonds and thereby increase the abundance of free flavonoids, which has also been reported in Asteraceae matrices processed under UAE conditions [14, 31].

The essential oil composition of *B. annua* reported by Porrello et al. [11], dominated by *β*‐myrcene and *β*‐pinene, further highlights the metabolic plasticity of this species. While those volatile terpenes represent a distinct biosynthetic domain, their coexistence with phenolic acids and flavonoids underscores the multifunctional chemical defense system of *B. annua*. The predominance of phenolic antioxidants over nonpolar metabolites in the present extracts is therefore coherent with both the polarity of methanol and the ecological roles attributed to phenolic compounds in stress tolerance and interspecies interactions.

The phytochemical pattern observed in *B. annua* places this species within the broader chemical framework of the *Bellis* genus, particularly with respect to the predominance of hydroxycinnamic acids and flavonoid glycosides such as chlorogenic acid and hyperoside, while previous phytochemical investigations have reported a comparatively lower contribution of triterpenoid saponins in *B. annua* relative to other congeners [32, 33]. These findings not only expand the current phytochemical knowledge of *B. annua* but also suggest that the species could serve as a valuable source of hydroxycinnamate and flavonoid antioxidants.

### 3.2 Antioxidant Activities of the Extracts

The antioxidant capacities of the *B. annua* methanolic extracts, evaluated through multiple in vitro assays, are summarized in Table 2. All extracts demonstrated notable reducing and radical‐scavenging potentials, although their efficiencies varied among assays. In the phosphomolybdenum test, the MAC‐ME extract exhibited the lowest EC_50_ value (1.56 mg/mL), followed closely by UAE‐ME (1.68 mg/mL) and SOE‐ME (1.76 mg/mL), indicating comparable total antioxidant capacities (*p *> 0.05). The reducing abilities measured by the CUPRAC and FRAP assays exhibited comparable patterns. In the CUPRAC test, SOE‐ME (0.78 mg/mL) and UAE‐ME (0.79 mg/mL) demonstrated the highest and statistically similar reducing capacities, while MAC‐ME (0.81 mg/mL) showed a slightly weaker yet comparable effect (*p *> 0.05). A similar tendency was observed in the FRAP assay, where SOE‐ME (0.66 mg/mL) and MAC‐ME (0.69 mg/mL) outperformed UAE‐ME (0.81 mg/mL), indicating that extraction conditions modestly influenced the electron‐donating potential of the extracts.

**TABLE 2:** Antioxidant activities of the methanol extracts from *B. annua*.

Assays	MAC‐ME	SOE‐ME	UAE‐ME	Trolox	EDTA
Phosphomolybdenum (EC_50_: mg/mL)	1.56 ± 0.10^*b*^	1.76 ± 0.09^*b*^	1.68 ± 0.34^*b*^	0.50 ± 0.05^*a*^	—
CUPRAC reducing power (EC_50_: mg/mL)	0.81 ± 0.004^*b*^	0.78 ± 0.01^*b*^	0.79 ± 0.003^*b*^	0.13 ± 0.02^*a*^	—
FRAP reducing power (EC_50_: mg/mL)	0.69 ± 0.003^*b*^	0.66 ± 0.03^*b*^	0.81 ± 0.03^*c*^	0.045 ± 0.004^*a*^	—
DPPH radical scavenging (IC_50_: mg/mL)	1.21 ± 0.005^*c*^	1.12 ± 0.004^*b*^	1.16 ± 0.01d	0.057 ± 0.002^*a*^	—
ABTS radical cation scavenging (IC_50_: mg/mL)	1.66 ± 0.03^*c*^	1.52 ± 0.01^*b*^	1.58 ± 0.01^*b*^	0.12 ± 0.02^*a*^	—
Ferrous ion chelating (IC_50_: mg/mL)	1.82 ± 0.01^*b*^	2.51 ±0.001^*c*^	1.85 ± 0.02^*b*^	—	0.035 ± 0.003^*a*^

Data are presented as mean ± standard deviation (SD) of three independent experiments (*n* = 3). Statistical differences were evaluated using one‐way ANOVA followed by Tukey's HSD post hoc test at *p *< 0.05.

Identical superscript letters within the same column indicate that the corresponding values are not significantly different at the *p *< 0.05 level.

Regarding free radical scavenging, the DPPH and ABTS assays confirmed the efficient hydrogen‐ or electron‐donating abilities of the extracts. SOE‐ME produced the lowest IC_50_ values in both DPPH (1.12 mg/mL) and ABTS (1.52 mg/mL) assays, suggesting a marginally stronger quenching capacity than MAC‐ME and UAE‐ME (*p *< 0.05). In contrast, the ferrous ion‐chelating activity followed a distinct trend, where MAC‐ME (1.82 mg/mL) and UAE‐ME (1.85 mg/mL) were significantly more active than SOE‐ME (2.51 mg/mL), though all were less potent than the reference chelator EDTA (0.035 mg/mL).

**FIGURE 3:**
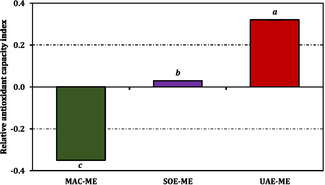
Relative antioxidant capacity index of the methanol extracts from *B. annua*. Data are presented as mean ± SD of three independent experiments (*n* = 3). Statistical differences were evaluated using one‐way ANOVA followed by Tukey's HSD post hoc test at *p *< 0.05.

**FIGURE 4:**
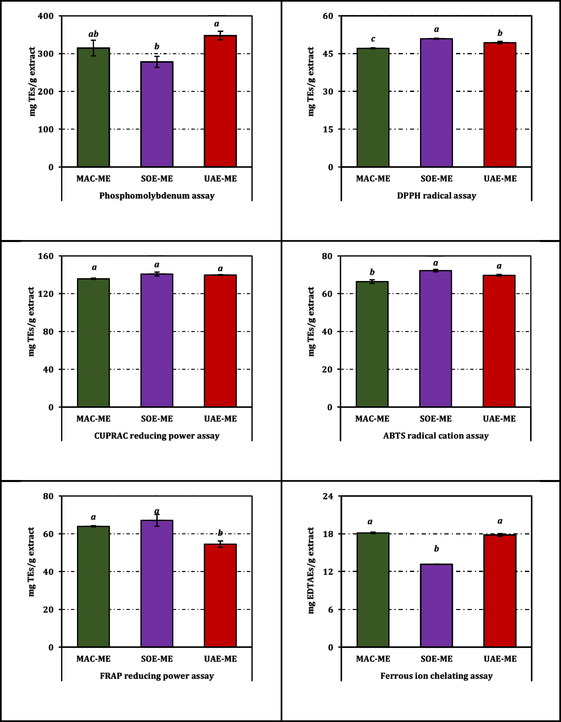
Antioxidant activity of the methanol extracts from *B. annua*. TEs and EDTAEs, trolox and ethylenediaminetetraacetic acid (disodium salt) equivalents, respectively. Data are presented as mean ± SD of three independent experiments (*n* = 3). Statistical differences were evaluated using one‐way ANOVA followed by Tukey's HSD post hoc test at *p *< 0.05.

When the data were integrated into a composite evaluation, the RACI values (Figure 3) revealed that UAE‐ME possessed the highest normalized antioxidant potential (0.32), followed by SOE‐ME (0.03) and MAC‐ME (−0.35). These findings align with the general trend observed in the individual assays, highlighting the overall consistency of the results. For visualization of the same data expressed in Trolox or EDTA equivalent units, readers are referred to Figure 4.

The current study provides the first comprehensive evaluation of the antioxidant potential of *B. annua*, revealing that its methanolic extracts possess consistent and multifaceted antioxidant activities across various assays. The observed outcomes, while species‐specific, align in part with those reported for other congeners within the *Bellis* genus, underscoring a general biochemical tendency of the group to accumulate phenolic compounds with electron‐ and hydrogen‐donating capabilities.

The parallel efficiency of *B. annua* extracts in both reducing and radical scavenging systems suggests the presence of multifunctional phenolics capable of acting through different antioxidant mechanisms. Comparable findings have been described in *B. perennis*, where methanolic extracts demonstrated notable scavenging effects on DPPH radicals, particularly when the plants were exposed to abiotic stress conditions that stimulated phenolic biosynthesis [34]. This supports the notion that *Bellis* species can dynamically regulate secondary metabolism in response to environmental cues, leading to enhanced antioxidant profiles.

In contrast, earlier reports using nonoptimized extraction protocols for *B. perennis* yielded considerably weaker DPPH scavenging activity [35]. The low activity levels in that study can likely be attributed to differences in solvent polarity, extraction yield, and the relatively crude nature of the tested fractions. Later optimization efforts confirmed that improved extraction parameters, particularly those maximizing flavonoid recovery, significantly enhance the antioxidant performance of *B. perennis* [36]. In this respect, the current findings for *B. annua* are in line with the optimized patterns observed in *B. perennis*, indicating that the phenolic–flavonoid balance rather than the extraction technique alone dictates the overall antioxidant potential.

When the antioxidant mechanisms are considered collectively, the relatively high reducing capacity and radical quenching efficiency of *B. annua* extracts indicate a prevalence of redox‐active constituents that can both terminate radical chain reactions and regenerate oxidized intermediates. The moderate chelating activity detected complements this view, implying that the extract can exert both preventive and chain‐breaking antioxidant functions. Such dual functionality has been previously described for *B. perennis* extracts [37], suggesting a conserved biochemical strategy among Bellis species to counter oxidative stress through multiple complementary mechanisms.

Integrating the current results with prior knowledge thus broadens the understanding of antioxidant diversity within the *Bellis* genus. The relatively balanced activities of B. annua across assays, together with its rich phenolic content reported in the accompanying phytochemical analysis, place this taxon among promising natural sources of multifunctional antioxidants. Considering the ecological and biochemical proximity between *B. annua* and *B. perennis*, it is plausible that similar biosynthetic pathways underlie their antioxidant potential. Future studies focusing on the isolation and structural elucidation of individual active compounds, as well as comparative in vivo validations, would provide deeper insight into the adaptive and pharmacological significance of these findings.

### 3.3 Enzyme Inhibitory Activities of the Extracts

The enzyme inhibitory potentials of the methanol extracts obtained from *B. annua* through different extraction techniques are summarized in Table 3. All extracts exhibited a concentration‐dependent inhibition toward AChE, tyrosinase, and *α*‐amylase, albeit with varying efficiencies across assays.

**TABLE 3:** Enzyme inhibition activity of the methanol extracts from *B. annua*.

Samples	AChE inhibition (IC_50_: mg/mL)	Tyrosinase inhibition (IC_50_: mg/mL)	*α* ‐Amylase inhibition (IC_50_: mg/mL)
MAC‐ME	1.46 ± 0.02^*b*^	1.41 ± 0.002^*d*^	1.54 ± 0.03^*b*^
SOE‐ME	1.65 ± 0.02^*c*^	1.30 ± 0.01^*b*^	1.83 ± 0.01^*c*^
UAE‐ME	1.71 ± 0.02^*c*^	1.37 ± 0.003^*c*^	1.63 ± 0.01^*b*^
Galanthamine	0.0025 ± 0.0003^*a*^	—	—
Kojic acid	—	0.084 ± 0.004^*a*^	—
Acarbose	—	—	0.81 ± 0.03^*a*^

Data are presented as mean ± standard deviation (SD) of three independent experiments (*n* = 3). Statistical differences were evaluated using one‐way ANOVA followed by Tukey's HSD post hoc test at *p *< 0.05.

Identical superscript letters within the same row indicate that the corresponding values are not significantly different at the *p *< 0.05 level.

For AChE inhibition, the most pronounced effect was recorded for the MAC‐ME extract, with an IC_50_ value of 1.46 mg/mL, followed by SOE‐ME (1.65 mg/mL) and UAE‐ME (1.71 mg/mL). Although these values are markedly higher than that of the reference compound galanthamine (0.0025 mg/mL), they still reflect a measurable inhibitory potential that may relate to the phenolic and flavonoid constituents identified in the extracts.

Regarding tyrosinase inhibition, SOE‐ME demonstrated the most effective activity (IC_50_ = 1.30 mg/mL), surpassing both UAE‐ME (1.37 mg/mL) and MAC‐ME (1.41 mg/mL). Despite being considerably weaker than kojic acid (IC_50_ = 0.084 mg/mL), the relatively low IC_50_ values observed among the extracts indicate a meaningful capacity to interfere with melanogenesis‐related enzyme activity.

In the *α*‐amylase assay, MAC‐ME again showed superior inhibition (IC_50_ = 1.54 mg/mL), while UAE‐ME (1.63 mg/mL) and SOE‐ME (1.83 mg/mL) followed closely. Although all extracts were less potent than acarbose (IC_50_ = 0.81 mg/mL), their moderate inhibitory effects suggest potential complementary activity in carbohydrate metabolism regulation.

**FIGURE 5:**
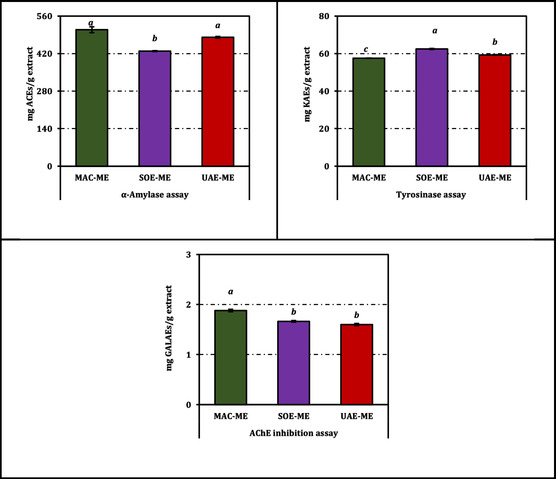
Enzyme inhibition activity of the methanol extracts from *B. annua*. ACEs, GALAEs and KAEs mean acarbose, galanthamine and kojic acid equivalents, respectively. Data are presented as mean ± SD of three independent experiments (*n* = 3). Statistical differences were evaluated using one‐way ANOVA followed by Tukey's HSD post hoc test at *p *< 0.05.

When considered as a whole, the comparative performance of the extracts indicates that the MAC method tends to yield slightly stronger inhibition of AChE and *α*‐amylase, whereas SOE is more favorable for tyrosinase inhibition. For a visual representation of the inhibition profiles expressed as positive control equivalents, readers are referred to Figure 5.

This study provides the first detailed account of the enzyme inhibitory properties of *B. annua*, thereby expanding the biochemical and pharmacological understanding of the genus *Bellis*. Although the inhibitory potencies of the methanol extracts were modest compared to standard drugs, the consistent inhibition of AChE, tyrosinase, and *α*‐amylase observed across all extraction techniques suggests a multifaceted biological potential. Such activities are of particular interest considering the growing demand for natural enzyme modulators that may provide safer and complementary alternatives to synthetic inhibitors used in neurodegenerative, dermatological, and metabolic disorders.

The cholinesterase inhibitory activity observed here parallels the findings reported for *B. perennis*, whose isolated apigenin derivatives also showed appreciable AChE inhibition in vitro [9]. The presence of these compounds in *B. annua* extracts implies a possible mechanistic similarity within the genus. Indeed, apigenin‐7‐glucoside has been proposed to interact with the catalytic anionic site of AChE through *π*–*π* stacking and hydrogen bonding, while chlorogenic acid is capable of modulating the peripheral anionic site, thereby reducing acetylcholine hydrolysis [5, 38]. Although glycosylation of apigenin tends to reduce inhibitory potency relative to its aglycone [39], the cumulative effect of multiple phenolic constituents may still account for the overall measurable inhibition seen in the present extracts.

Tyrosinase inhibition, which was most pronounced in the Soxhlet extract, aligns with previous evidence linking chlorogenic acid and hyperoside to melanogenesis control. Chlorogenic acid has been reported to chelate the copper ions in the enzyme's active site and impede the oxidation of L‐DOPA [40], while apigenin‐7‐glucoside was shown to significantly suppress melanin synthesis in B16F10 melanoma cells [41]. Likewise, hyperoside, a major flavonoid detected in *B. annua*, exhibits tyrosinase inhibition through antioxidant‐mediated modulation of melanogenic pathways [42]. Collectively, these observations support the notion that the moderate tyrosinase inhibitory activity of *B. annua* extracts could be attributed to synergistic interactions among its polyphenolic constituents rather than the dominance of a single molecule.

The inhibition of *α*‐amylase recorded for *B. annua* also provides a plausible biochemical link to the hypoglycemic potential reported for *B. perennis*, which was shown to stimulate GLUT4 translocation and reduce blood glucose levels in vivo [10]. Polyphenols such as hyperoside and chlorogenic acid are well known to attenuate carbohydrate digestion and absorption through binding to the active site of *α*‐amylase and altering its catalytic conformation [43, 44]. Therefore, the *α*‐amylase inhibitory effect observed here likely complements the antioxidant and anti‐diabetic mechanisms associated with these compounds, highlighting *B. annua* as a promising candidate for metabolic health applications.

When extraction techniques were compared, the higher AChE and *α*‐amylase inhibition observed in the macerated extract was consistent with the preservation of heat‐labile flavonoids, whereas the stronger tyrosinase inhibition exhibited by the Soxhlet extract was associated with its higher chlorogenic acid and phenolic acid content, as quantitatively determined by LC–ESI–MS/MS. This variation emphasizes the importance of extraction optimization in maximizing enzyme‐specific bioactivity profiles.

The current findings demonstrate that *B. annua* methanol extracts, although less potent than synthetic reference inhibitors, exhibit consistent and biologically relevant multienzyme inhibitory activities. These effects are plausibly linked to its phenolic and flavonoid composition, particularly chlorogenic acid, hyperoside, hesperidin, and apigenin‐7‐glucoside. The observed trends are coherent with those documented for related *Bellis* species, suggesting that this genus may represent an underexplored source of natural enzyme inhibitors with potential applications in managing neurodegenerative, pigmentary, and metabolic disorders.

### 3.4 Correlations among Phenolic Compounds and Assays

The Pearson correlation matrix (Table 4) provided a detailed overview of the interrelationships among phenolic compounds, total phenolic/flavonoid contents, antioxidant assays, and enzyme inhibition activities. As expected, most antioxidant assays exhibited strong positive correlations with each other, indicating that the extracts shared consistent redox behavior across different evaluation systems. Notably, DPPH, ABTS, and CUPRAC assays were highly correlated, supporting their mutual sensitivity to electron‐donating antioxidants. In contrast, FRAP showed weaker and occasionally inverse associations with these assays, implying that its reaction mechanism may favor distinct reducing agents compared to the other systems.

**TABLE 4:** Correlations among phenolic compounds and assays.

	TAP	DPPH	ABTS	CUPRAC	FRAP	FICA	AChEIA	TIA	AAIA
DPPH	−0.421	—	—	—	—	—	—	—	—
ABTS	−0.372	0.944	—	—	—	—	—	—	—
CUPRAC	−0.280	0.871	0.903	—	—	—	—	—	—
FRAP	−0.785	0.131	0.139	−0.147	—	—	—	—	—
FICA	0.803	−0.838	−0.824	−0.647	−0.625	—	—	—	—
AChEIA	−0.196	−0.796	−0.768	−0.807	0.454	0.350	—	—	—
TIA	−0.610	0.960	0.932	0.793	0.394	−0.954	−0.611	—	—
AAIA	0.656	−0.954	−0.911	−0.824	−0.373	0.950	0.590	−0.990	—
Total flavonoid	0.795	−0.181	−0.167	0.148	−0.962	0.649	−0.399	−0.422	0.390
Total phenolic	0.455	−0.122	0.155	0.057	−0.307	0.211	−0.172	−0.153	0.233
RACI	0.429	0.502	0.644	0.691	−0.563	−0.100	−0.844	0.341	−0.303
Chlorogenic acid	−0.087	−0.849	−0.830	−0.867	0.333	0.454	0.977	−0.698	0.686
Hyperoside	0.692	−0.878	−0.829	−0.607	−0.549	0.965	0.452	−0.956	0.929
Hesperidin	0.891	−0.714	−0.713	−0.524	−0.715	0.972	0.166	−0.866	0.866
Apigenin 7‐glucoside	0.883	−0.682	−0.682	−0.462	−0.789	0.970	0.118	−0.855	0.847
Caffeic acid	0.768	−0.859	−0.830	−0.633	−0.592	0.992	0.393	−0.958	0.945
Luteolin 7‐glucoside	0.913	−0.297	−0.323	−0.088	−0.952	0.769	−0.324	−0.545	0.543
Protocatechuic acid	0.510	−0.953	−0.879	−0.845	−0.101	0.819	0.719	−0.907	0.918
Kaempferol	0.935	−0.561	−0.549	−0.332	−0.861	0.919	−0.045	−0.760	0.761
Verbascoside	0.701	0.293	0.300	0.465	−0.837	0.254	−0.804	0.044	−0.031
4‐Hydroxybenzoic acid	0.775	0.121	0.103	0.319	−0.941	0.433	−0.687	−0.149	0.150
Apigenin	0.909	−0.263	−0.295	−0.074	−0.955	0.746	−0.357	−0.517	0.520
Quercetin	0.140	0.723	0.742	0.881	−0.430	−0.330	−0.901	0.570	−0.587
Gallic acid	−0.297	−0.710	−0.695	−0.792	0.555	0.231	0.982	−0.508	0.499
p‐Coumaric acid	0.274	0.672	0.672	0.819	−0.629	−0.195	−0.957	0.460	−0.465
Rosmarinic acid	0.783	0.023	−0.048	0.201	−0.926	0.518	−0.581	−0.245	0.232
Ferulic acid	0.869	−0.063	−0.085	0.121	−0.961	0.594	−0.544	−0.330	0.338
Luteolin	0.856	−0.058	−0.092	0.127	−0.963	0.591	−0.543	−0.327	0.330
2,5‐Dihydroxybenzoic acid	0.646	−0.928	−0.940	−0.787	−0.430	0.967	0.559	−0.989	0.975
Syringic acid	−0.146	−0.801	−0.697	−0.668	0.353	0.378	0.951	−0.622	0.588
Vanillin	−0.433	0.953	0.994	0.920	0.172	−0.851	−0.742	0.949	−0.941
Sinapic acid	0.901	−0.199	−0.248	−0.032	−0.937	0.701	−0.411	−0.456	0.459
(−)‐Epicatechin	0.848	−0.006	−0.034	0.180	−0.935	0.545	−0.585	−0.269	0.273

Data show the Pearson Correlation Coefficients between the parameters. TAP, total antioxidant activity by phosphomolybdenum method. AAIA, AChEIA, and TIA, *α*‐amylase, acetylcholinesterase, and tyrosinase inhibition activities, respectively. ABTS and DPPH, ABTS and DPPH radical scavenging activities, respectively. CUPRAC and FRAP, CUPRAC and FRAP reducing power potential; respectively. FICA, Ferrous ion chelating activity.

Ferrous ion chelating activity (FICA) demonstrated a strong positive correlation with total flavonoid content (*r* = 0.649) and TAP values (*r* = 0.803), suggesting that metal‐binding activity in *B. annua* extracts was largely attributable to flavonoid‐type constituents. Among enzyme‐related parameters, tyrosinase inhibition (TIA) displayed robust positive correlations with radical scavenging assays (*r* = 0.960 with DPPH; *r* = 0.932 with ABTS), while AChE inhibition (AChEIA) was inversely related to antioxidant capacities (*r* = −0.796 to −0.807 versus DPPH and CUPRAC), highlighting the distinct mechanisms governing oxidative and enzymatic interactions.

Regarding individual metabolites, several phenolic acids and flavonoids exhibited strong contributions to both antioxidant and enzyme inhibition outcomes. Caffeic acid, hesperidin, apigenin 7‐glucoside, and hyperoside were among the most influential constituents, showing near‐perfect correlations with FICA (*r* = 0.965–0.992) and inverse correlations with DPPH and ABTS (*r* = −0.682 to −0.859). Similarly, kaempferol, luteolin, and ferulic acid were strongly associated with TAP activity (*r* > 0.85), confirming their role as electron donors. Conversely, compounds such as vanillin and *p*‐coumaric acid displayed significant positive relationships with radical scavenging assays (*r* up to 0.994), but negative or negligible correlations with AChEIA, suggesting specificity toward oxidative pathways rather than enzymatic modulation.

The RACI showed consistent alignment with CUPRAC and ABTS assays (*r* = 0.691 and 0.644, respectively), validating its reliability as a composite measure of overall antioxidant strength. Collectively, these correlations underscore that the antioxidant behavior of *B. annua* extracts is governed by synergistic interactions between flavonoid‐rich components and phenolic acids, each contributing selectively to radical scavenging, reducing power, and enzyme inhibition profiles.

## 4 Conclusions

This study compared methanol extracts obtained from *B. annua* using MAC, Soxhlet, and ultrasound‐assisted methods and demonstrated the effects of different extraction methods on the chemical composition and biological activity of the plant. Total phenolic contents of the extracts were found to be quite similar, but some differences were detected for individual compounds. The UAE‐ME method contained higher amounts of flavonoids, while the MAC‐ME method was enriched in chlorogenic and gallic acids. These chemical differences were consistent with the results of antioxidant and enzyme inhibition tests.

All extracts exhibited significant antioxidant activity, with reducing and radical scavenging powers generally close to each other. According to RACI analysis results, the UAE‐ME extract had the highest antioxidant capacity. This was attributed to its high flavonoid level and phenolic diversity. Different trends were observed for enzyme inhibition depending on the methods: the MAC‐ME extract showed a more pronounced effect on AChE and *α*‐amylase, while the SOE‐ME extract inhibited tyrosinase more strongly. Although the effects of all three extracts were lower than those of synthetic standards, these levels of activity suggest that *B. annua* can be considered a naturally occurring antioxidant and enzyme regulator.

Correlation analyses showed that antioxidant activity is mediated by the combined effects of flavonoids and hydroxycinnamic acids, while enzyme inhibition varies depending on the structural properties of the components. The agreement of RACI values with individual tests supports the reliability of this assessment approach.

In conclusion, *B. annua* is a plant species rich in phenolics but has been poorly studied. The extraction method is a determining factor in the amount and diversity of these compounds. Future studies focusing on determining appropriate extraction conditions, examining the structure–activity relationships of prominent phenolics, and validating laboratory results in biological systems will contribute to understanding the true biological and therapeutic value of the plant.

## Supporting Information

Additional supporting information can be found online in the Supporting Information section.

## Author Contributions

**Fatma Ozlem Kargin Solmaz**: investigation, conceptualization, data curation, formal analysis, methodology, software, validation, visualization. **Cengiz Sarikurkcu**: investigation, methodology, data curation, formal analysis, writing original draft, writing – review and editing. **Bektaş Tepe**: data curation, writing original draft, writing – review and editing.

## Funding

This study was supported by the Scientific Research Projects Coordination Unit of Afyonkarahisar Health Sciences University through project number (25.GENEL.015).

## Conflicts of Interest

The authors declare no conflicts of interest.

## Supporting information

Supplementary Material

## Data Availability

The data that support the findings of this study are available from the corresponding author upon reasonable request.

## References

[1] R. Apak , K. Güçlü , M. Özyürek , S. E. Karademir , and E. Erça , “The Cupric Ion Reducing Antioxidant Capacity and Polyphenolic Content of Some Herbal Teas,” International Journal of Food Sciences and Nutrition 57 (2009): 292–304. 10.1080/09637480600798132 17135020

[2] R. Apak , K. Güçlü , M. Özyürek , and S. E. Karademir , “Novel Total Antioxidant Capacity Index for Dietary Polyphenols and Vitamins C and E, Using Their Cupric Ion Reducing Capability in the Presence of Neocuproine: CUPRAC Method,” Journal of Agricultural and Food Chemistry 52 (2004): 7970–7981. 15612784 10.1021/jf048741x

[3] R. Apak , K. Güçlü , M. Özyürek , and S. E. Çelik , “Mechanism of Antioxidant Capacity Assays and the CUPRAC (cupric Ion Reducing Antioxidant Capacity) Assay,” Microchimica Acta 160 (2008): 413–419.

[4] B. Ou , M. Hampsch‐Woodill , and R. L. Prior , “Development and Validation of an Improved Oxygen Radical Absorbance Capacity Assay Using Fluorescein as the Fluorescent Probe,” Journal of Agricultural and Food Chemistry 49 (2001): 4619–4626. 11599998 10.1021/jf010586o

[5] G. Oboh , O. M. Agunloye , A. J. Akinyemi , A. O. Ademiluyi , and S. A. Adefegha , “Comparative Study on the Inhibitory Effect of Caffeic and Chlorogenic Acids on Key Enzymes Linked to Alzheimer's Disease and Some Pro‐Oxidant Induced Oxidative Stress in Rats’ Brain‐In Vitro,” Neurochemical Research 38 (2013): 413–419. 23184188 10.1007/s11064-012-0935-6

[6] M. B. Colovic , D. Z. Krstic , T. D. Lazarevic‐Pasti , A. M. Bondzic , and V. M. Vasic , “Acetylcholinesterase Inhibitors: Pharmacology and Toxicology,” Current Neuropharmacology 11 (2013): 315–335. 24179466 10.2174/1570159X11311030006 PMC3648782

[7] N. Kaur , V. Kumar , S. K. Nayak , P. Wadhwa , P. Kaur , and S. K. Sahu , “Alpha‐amylase as Molecular Target for Treatment of Diabetes Mellitus: A Comprehensive Review,” Chemical Biology & Drug Design 98 (2021): 539–560. 34173346 10.1111/cbdd.13909

[8] T.‐S. Chang , “An Updated Review of Tyrosinase Inhibitors,” International Journal of Molecular Sciences 10 (2009): 2440–2475. 19582213 10.3390/ijms10062440 PMC2705500

[9] T. H. C. Marques , C. H. S. De Melo , R. B. F. De Carvalho , et al., “Phytochemical Profile and Qualification of Biological Activity of an Isolated Fraction of Bellis Perennis,” Biological Research 46 (2013): 231–238. 24346069 10.4067/S0716-97602013000300002

[10] R. Haselgrübler , V. Stadlbauer , F. Stübl , et al., “Insulin Mimetic Properties of Extracts Prepared from Bellis Perennis,” Molecules 23 (2018): 2605. 30314325 10.3390/molecules23102605 PMC6222741

[11] A. Porrello , N. Badalamenti , V. Ilardi , and M. Bruno , “Chemical Composition of the Essential Oil of Different Parts of Bellis annua L. (Asteraceae) Collected in Sicily (Italy),” Natural Product Research (In press) (2025): 1–7, 10.1080/14786419.2025.2550013. 40844433

[12] J. Azmir , I. S. M. Zaidul , M. M. Rahman , et al., “Techniques for Extraction of Bioactive Compounds from Plant Materials: A Review,” Journal of Food Engineering 117 (2013): 426–436.

[13] K. Vilkhu , R. Mawson , L. Simons , and D. Bates , “Applications and Opportunities for Ultrasound Assisted Extraction in the Food Industry—A Review,” Innovative Food Science & Emerging Technologies 9 (2008): 161–169.

[14] F. Chemat , N. Rombaut , A.‐G. Sicaire , A. Meullemiestre , A.‐S. Fabiano‐Tixier , and M. Abert‐Vian , “Ultrasound Assisted Extraction of Food and Natural Products. Mechanisms, Techniques, Combinations, Protocols and Applications. A Review,” Ultrasonics Sonochemistry 34 (2017): 540–560. 27773280 10.1016/j.ultsonch.2016.06.035

[15] L. Shen , S. Pang , M. Zhong , et al., “A Comprehensive Review of Ultrasonic Assisted Extraction (UAE) for Bioactive Components: Principles, Advantages, Equipment, and Combined Technologies,” Ultrasonics Sonochemistry 101 (2023): 106646. 37862945 10.1016/j.ultsonch.2023.106646 PMC10594638

[16] A. Carreira‐Casais , P. Otero , P. Garcia‐Perez , et al., “Benefits and Drawbacks of Ultrasound‐Assisted Extraction for the Recovery of Bioactive Compounds from Marine Algae,” International Journal of Environmental Research and Public Health 18 (2021): 9153. 34501743 10.3390/ijerph18179153 PMC8431298

[17] T. P. Vo , T. V. Pham , T. N. H. Tran , et al., “Ultrasonic‐Assisted and Microwave‐Assisted Extraction of Phenolics and Terpenoids from *Abelmoschus Sagittifolius* (Kurz) Merr Roots Using Natural Deep Eutectic Solvents,” ACS Omega 8 (2023): 29704–29716. 37599925 10.1021/acsomega.3c03929 PMC10433328

[18] Q. D. Do , A. E. Angkawijaya , P. L. Tran‐Nguyen , et al., “Effect of Extraction Solvent on Total Phenol Content, Total Flavonoid Content, and Antioxidant Activity of Limnophila Aromatica,” Journal of Food and Drug Analysis 22 (2014): 296–302. 28911418 10.1016/j.jfda.2013.11.001 PMC9354875

[19] J. Dai and R. J. Mumper , “Plant Phenolics: Extraction, Analysis and Their Antioxidant and Anticancer Properties,” Molecules 15 (2010): 7313–7352. 20966876 10.3390/molecules15107313 PMC6259146

[20] M. Cittan and A. Çelik , “Development and Validation of an Analytical Methodology Based on Liquid Chromatography–Electrospray Tandem Mass Spectrometry for the Simultaneous Determination of Phenolic Compounds in Olive Leaf Extract,” Journal of Chromatographic Science 56 (2018): 336–343. 29373655 10.1093/chromsci/bmy003

[21] T. Sun and S. A. Tanumihardjo , “An Integrated Approach to Evaluate Food Antioxidant Capacity,” Journal of Food Science 72 (2007): R159–R165. 18034745 10.1111/j.1750-3841.2007.00552.x

[22] G. Zengin , A. Cvetanović , U. Gašić , et al., “Chemical Composition and Bio‐Functional Perspectives of Erica Arborea L. Extracts Obtained by Different Extraction Techniques: Innovative Insights,” Industrial Crops and Products 142 (2019): 111843.

[23] G. Zengin , M. C. Uren , M. S. Kocak , et al., “Antioxidant and Enzyme Inhibitory Activities of Extracts from Wild Mushroom Species from Turkey,” International Journal of Medicinal Mushrooms 19 (2017): 327–336. 28605321 10.1615/IntJMedMushrooms.v19.i4.30

[24] M. S. Ozer , B. Kirkan , C. Sarikurkcu , et al., “Onosma Heterophyllum: Phenolic Composition, Enzyme Inhibitory and Antioxidant Activities,” Industrial Crops and Products 111 (2018): 179–184.

[25] M. Scognamiglio , E. Buommino , L. Coretti , et al., “Phytochemical Investigation and Antimicrobial Assessment of Bellis Sylvestris Leaves,” Phytochemistry Letters 17 (2016): 6–13.

[26] G. Cingöz and F. P. Karakaş , “The Effects of Nutrient and Macronutrient Stress on Certain Secondary Metabolite Accumulations and Redox Regulation in Callus Cultures of Bellis Perennis L.,” Turkish Journal of Biology 40 (2016): 1328–1335.

[27] M. Scognamiglio , A. Esposito , B. D’Abrosca , et al., “Isolation, Distribution and Allelopathic Effect of Caffeic Acid Derivatives from Bellis Perennis L.,” Biochemical Systematics and Ecology 43 (2012): 108–113.

[28] Y. Wang , Z. Xu , Y. Huang , et al., “Extraction, Purification, and Hydrolysis Behavior of Apigenin‐7‐O‐Glucoside from Chrysanthemum Morifolium Tea,” Molecules 23 (2018): 2933. 30424020 10.3390/molecules23112933 PMC6278536

[29] T. Schöpke , K. Hiller , V. Wray , and M. Nimtz , “Application of MS‐MS for the Rapid, Comparative Analysis of Saponin Mixtures as Exemplified by the Deacylated and Partially Deacylated Triterpenoid Saponins of *Bellis Annua* ,” Planta Medica 62 (1996): 336–340. 8792666 10.1055/s-2006-957897

[30] T. Schöpke , K. Hiller , V. Wray , M. Nimtz , K. Yamasaki , and R. Kasai , “Triterpenoid Saponins from *Bellis Sylvestris* . 2. Structures of Partially Deacylated Saponins,” Journal of Natural Products 59 (1996): 355–359. 8699180 10.1021/np9600715

[31] F. P. Karakas , G. Sahin , A. U. Turker , and S. K. Verma , “Impacts of Heavy Metal, High Temperature, and UV Radiation Exposures on Bellis Perennis L. (common Daisy): Comparison of Phenolic Constituents and Antioxidant Potential (enzymatic and Non‐Enzymatic),” South African Journal of Botany 147 (2022): 370–379.

[32] O. Ceylan , A. Ugur , and N. Sarac , “In Vitro Antimicrobial, Antioxidant, Antibiofilm and Quorum Sensing Inhibitory Activities of Bellis Perennis L,” Journal of BioScience and Biotechnology 2014 (2014): 35–42.

[33] O. Bouallag , S. Zeghichi‐Hamri , M. Bachir‐Bey , et al., “Optimization of Phenolic Compounds Extraction from Bellis Perennis Flowers and Assessment for Antioxidant Properties,” The Annals of the University Dunarea de Jos of Galati. Fascicle VI‐Food Technology 46 (2022): 125–139.

[34] N. Kavalcioğrlu , L. Açık , F. Demirci , B. Demirci , H. Demir , and K. H. C. Başer , “Biological Activities of Bellis Perennis Volatiles and Extracts,” Natural Product Communications 5 (2010): 1934578X1000500134. 20184041

[35] E. S. Istifli and C. Sarıkürkcü , “Assessment of Apigenin‐7‐Glucoside and Luteolin‐7‐Glucoside as Multi‐Targeted Agents against Alzheimer's Disease: A Molecular Docking Study,” International Journal of Plant Based Pharmaceuticals 1 (2021): 56–64.

[36] E. Witkowska‐Banaszczak , V. Krajka‐Kuźniak , and K. Papierska , “The Effect of Luteolin 7‐Glucoside, Apigenin 7‐Glucoside and Succisa Pratensis Extracts on NF‐κB Activation and α‐Amylase Activity in HepG2 Cells,” Acta Biochimica Polonica 67 (2020): 41–47. 32129972 10.18388/abp.2020_2894

[37] M. M. de Freitas , P. R. Fontes , P. M. Souza , et al., “Extracts of Morus Nigra L. Leaves Standardized in Chlorogenic Acid, Rutin and Isoquercitrin: Tyrosinase Inhibition and Cytotoxicity,” PloS One 11 (2016): e0163130. 27655047 10.1371/journal.pone.0163130 PMC5031429

[38] N. N. Bouzaiene , F. Chaabane , A. Sassi , L. Chekir‐Ghedira , and K. Ghedira , “Effect of Apigenin‐7‐Glucoside, Genkwanin and Naringenin on Tyrosinase Activity and Melanin Synthesis in B16F10 Melanoma Cells,” Life Sciences 144 (2016): 80–85. 26656314 10.1016/j.lfs.2015.11.030

[39] E. Ersoy , E. E. Ozkan , M. Boga , M. A. Yilmaz , and A. Mat , “Anti‐Aging Potential and Anti‐Tyrosinase Activity of Three Hypericum Species with Focus on Phytochemical Composition by LC–MS/MS,” Industrial Crops and Products 141 (2019): 111735.

[40] A. Albadawi , A. Elkashef , M. Nofel , et al., “In Vitro and In Silico Antioxidant and Antidiabetic Properties of Zinc Oxide‐Chlorogenic Acid Nanoparticles Composite,” Alexandria Journal of Science and Technology 2 (2024): 1–15.

[41] H. Shen , J. Wang , J. Ao , et al., “Structure‐Activity Relationships and the Underlying Mechanism of α‐Amylase Inhibition by Hyperoside and Quercetin: Multi‐Spectroscopy and Molecular Docking Analyses,” Spectrochimica Acta Part A: Molecular and Biomolecular Spectroscopy 285 (2023): 121797. 36115306 10.1016/j.saa.2022.121797

[42] M. S. Kocak , C. Sarikurkcu , M. Cengiz , S. Kocak , M. C. Uren , and B. Tepe , “Salvia Cadmica: Phenolic Composition and Biological Activity,” Industrial Crops and Products 85 (2016): 204–212.

[43] B. Tepe , C. Sarikurkcu , S. Berk , A. Alim , and H. A. Akpulat , “Chemical Composition, Radical Scavenging and Antimicrobial Activity of the Essential Oils of Thymus boveii and Thymus hyemalis,” Records of Natural Products 5 (2011): 208–220.

[44] C. Sarikurkcu , M. Locatelli , A. Mocan , G. Zengin , and B. Kirkan , “Phenolic Profile and Bioactivities of Sideritis Perfoliata L.: The Plant, Its Most Active Extract, and Its Broad Biological Properties,” Frontiers in Pharmacology 10 (2020): 1642. 32116669 10.3389/fphar.2019.01642 PMC7034418

